# The Extrusion Process as an Alternative for Improving the Biological Potential of Sorghum Bran: Phenolic Compounds and Antiradical and Anti-Inflammatory Capacity

**DOI:** 10.1155/2016/8387975

**Published:** 2016-09-25

**Authors:** Norma Julieta Salazar Lopez, Guadalupe Loarca-Piña, Rocío Campos-Vega, Marcela Gaytán Martínez, Eduardo Morales Sánchez, J. Marina Esquerra-Brauer, Gustavo A. Gonzalez-Aguilar, Maribel Robles Sánchez

**Affiliations:** ^1^Departamento de Investigación y Posgrado en Alimentos, Universidad de Sonora, 83000 Hermosillo, SON, Mexico; ^2^Departamento de Investigación y Posgrado en Alimentos, Facultad de Química, Universidad Autónoma de Querétaro, 76010 Santiago de Querétaro, QRO, Mexico; ^3^Instituto Politécnico Nacional, Centro de Investigación en Ciencia Aplicada y Tecnología Avanzada, 76090 Santiago de Querétaro, QRO, Mexico; ^4^Centro de Investigación en Alimentación y Desarrollo, A.C., 83304 Hermosillo, SON, Mexico

## Abstract

Approximately 80% of sorghum phenolic compounds are linked to arabinoxylans by ester bonds, which are capable of resisting the digestion process in the upper gastrointestinal tract, compromising their bioaccessibility and biological potential. The aim of this study was to evaluate the effect of the extrusion process on the content of phenolic compounds in sorghum bran and its impact on phenolic compounds and antiradical and anti-inflammatory capacity. Results revealed that the extrusion process increased total phenol content in sorghum bran compared to nonextruded sorghum, particularly for extrusion at 180°C with 20% moisture content (2.0222 ± 0.0157 versus 3.0729 ± 0.0187 mg GAE/g +52%), which positively affected antiradical capacity measured by the DPPH and TEAC assays. The percentage of inhibition of nitric oxide (NO) production by RAW cells due to the presence of extruded sorghum bran extract was significantly higher than that of nonextruded sorghum bran extract (90.2 ± 1.9% versus 76.2 ± 1.3%). The results suggest that extruded sorghum bran could be used as a functional ingredient and provide advantages to consumers by reducing diseases related to oxidative stress and inflammation.

## 1. Introduction

Sorghum, the fifth most important cereal grown in the world, is resistant to semiarid climates, gluten-free, and a good source of phytochemical compounds that have been associated with antioxidant, anti-inflammatory, and antiproliferative capacities [1–4]. The biological potential of sorghum has been related to the presence of different hydroxycinnamic acids (HCAs) such as ferulic, *ρ*-coumaric, caffeic, and sinapic acids.

However, much of the biological potential of sorghum is not used by biological systems due to the structural properties of their phenolic acids. Approximately 80% of these compounds are linked by ester bonds to arabinoxylans (ARAs), located mainly in the cell walls of the pericarp and the aleurone layer [5, 6]. The linkage between HCAs and ARAs restricts their bioaccessibility and further bioavailability because ARAs are resistant to the digestion process in the upper gastrointestinal tract, which compromises their absorption. Therefore, it is necessary to find processes that increase the bioaccessibility of the phenolic compounds prior to intake of this cereal.

The structure of arabinoxylans can be hydrolyzed by chemical processes, thermal processes, fermentation, enzymatic action, or a combination of these processes [7–11]. Rosa et al. [12] reported an increase in bioaccessibility of 86% of ferulic acid from the aleurone layer in wheat with the use of xylanase and ferulic esterase. Bartolomé and Gómez-Cordovés [13] found approximately 70% and 5% of ferulic acid and *ρ*-coumaric acid, respectively, released from barley using commercial enzyme preparations. However, enzymatic processes in sorghum can be more complicated than in wheat or barley because the sorghum arabinoxylan structure is more substituted, which is more difficult for the enzyme to gain access to the attached sites between ferulic acid and arabinose; therefore, chaperone enzymes are necessary [14–17]. Cardoso et al. [11] and Afify et al. [18] reported a loss of phenolic compound content and antioxidant capacity after the traditional processes of wet cooking and soaking of sorghum. On the other hand, Zielinski et al. [19] and Gumul and Korus [20] reported an increase in total phenolic content and hydroxycinnamic acids, mainly ferulic acid, in barley, rice, oats, wheat, and rye, after extrusion processes. These studies reveal that extrusion is a promising process in the production of functional foods based on cereals [19, 20].

Extrusion consists of heat and mechanical treatments under different conditions of low moisture, shear, and high pressure by producing structural alterations and changes in functional properties in a short time [21]. The effect of extrusion on the content of nutrients and nonnutritious components such as phenolic compounds depends on the process conditions and the food matrix [21]. The aim of this study was to evaluate the effect of the extrusion process under different temperature and moisture levels on the content of phenolic compounds and antiradical and anti-inflammatory capacity in sorghum bran.

## 2. Materials and Methods

### 2.1. Materials and Chemicals

Antibiotic-antimycotic, fetal bovine serum, sodium pyruvate, and Dulbecco's modified Eagle's medium (DMEM) were obtained from Gibco (Grand Island, NY, USA). All other reagents were purchased from Sigma-Aldrich (Saint Louis, Missouri, USA).

### 2.2. Sorghum Sample Preparation

Sorghum grains (*Sorghum bicolor* L. Moench), unpigmented variety (UDG110), were provided by the Produce Foundation, Mexico. The sorghum grains were decorticated using abrasive discs for 6 min and further ground using Pulvex 200 mill to pass through a 0.4 mm sieve. The sorghum bran was stored at −20°C until analysis.

### 2.3. Extrusion Procedure

The sorghum bran was allowed to hydrate for 8 h (20% and 30%) before extrusion and processed in an extruder (prototype) with a single screw with length of 45 cm and two jackets with length of 15 and 10 cm. The temperature of the first jacket was controlled to 60°C, while that of the second jacket was set to 110°C or 180°C. The screw speed was 15 rpm and the die diameter was 5 mm. The extrudates were dried in an oven at 60°C for 6 h. The dried products were ground and sieved with a 0.4 mm sieve and stored at −20°C until analysis.

### 2.4. Preparation of Sorghum Bran Extracts

Extruded sorghum bran (EB) or nonextruded sorghum bran (NEB) extracts were prepared as follows: 1 g of each sample was mixed with 15 mL of 80% aqueous methanol, sonicated for 1 h (100 W power output), and centrifuged at 1500 ×g for 15 min [6]. The supernatants were separated, and the residues were extracted twice for 30 min. Extracts were filtered through Whatman number 1 paper and evaporated to dryness in a rotary evaporator at 35°C, and samples were redissolved in 5 mL of 50% methanol for the analysis of total phenolic content, phenolic acid content, and antiradical capacity. The extracts were lyophilized and redissolved in DMSO for the cell culture tests.

### 2.5. Quantification of Phenolic Acids by UHPLC-DAD

The phenolic acid content in EB and NEB extracts was quantified using UHPLC system (Agilent Technologies, Germany) with a diode array detector. The separation was conducted on a Zorbax Eclipse Plus C18 rapid resolution column (50 mm × 2.1 mm i.d., 1.8 *μ*m particle size). Column temperature was set to 30°C. A binary phase solvent system was used, A (0.1% acetic acid/water) and B (0.1% acetic acid/methanol), at a flow rate of 0.7 mL/min. The solvent gradient was as follows: initial 91% of A and 9% of B; 0–11 min, 9% to 14% B; and 11–15 min, 15% B. Detection of the acids was performed at 280 nm, and their quantitation was performed with curves established using external standards of caffeic, *ρ*-coumaric, ferulic, and sinapic acids. The results were expressed as *μ*g phenolic acid per gram of dry weight [22].

### 2.6. Determination of Total Phenolic Content

The total phenolic content of the EB and NEB extracts was determined by the colorimetric method at 765 nm using the Folin–Ciocalteu reagent [23]. The results were expressed as mg of gallic acid equivalents (GAE) per gram of dry weight.

### 2.7. Antiradical Capacity

#### 2.7.1. DPPH Assay

This assay is based on the measurement of the scavenging ability of antioxidants towards the stable radical DPPH relative to the DPPH scavenging ability of the water-soluble vitamin E analogue Trolox. Briefly, 3.9 mL aliquots of DPPH (0.0634 mM) solution were added to the test tubes, and 0.1 mL of sorghum bran extracts (EB or NEB) or Trolox standards (0 to 20 *μ*M range) were added and shaken vigorously. The tubes were allowed to stand at 25°C for 60 min. A control reaction was prepared as above without any extract, and methanol was used for the baseline correction. Changes in the absorbance of the samples were measured at 515 nm. Radical scavenging activity was expressed as the percent inhibition. The final DPPH values were calculated by using a regression equation between the Trolox concentration and the percent inhibition and were expressed as micromoles of Trolox equivalents per gram of dry weight [24].

#### 2.7.2. Trolox Equivalent Antioxidant Capacity (TEAC)

The antiradical potential was determined using 2,2′-azino-bis(3-ethylbenzothiazoline-6-sulfonic acid) diammonium salt (ABTS) [25]. This assay is based on the ability of antioxidants to scavenge the blue-green ABTS^•+^ radical cation, relative to the ABTS^•+^ scavenging ability of the water-soluble vitamin E analogue Trolox. The ABTS^•+^ radical cation was generated by the interaction of 5 mL of 7 mM ABTS solution and 88 *μ*L of 140 mM K_2_S_2_O_8_ solution. The working solution was prepared with 1 mL of the active radical and 88 mL of ethanol for initial absorbance of 0.70 ± 0.2 at 734 nm using a Cary 50 Varian Spectrophotometer. After the addition of 2.9 mL of ABTS^•+^ solution to 0.1 mL of each extract or Trolox standards (0 to 20 *μ*M range), the absorbance was monitored exactly 1 and 30 min after the initial mixing until the absorbance was stable. The percentage of absorbance inhibition at 734 nm was calculated and plotted as a function of that obtained for the extracts and the standard reference (Trolox). The final TEAC values were calculated by using a regression equation between the Trolox concentration and the percentage inhibition and expressed as micromoles of Trolox equivalents per gram of dry weight.

### 2.8. Anti-Inflammatory Capacity

#### 2.8.1. Cell Culture

Mouse macrophage cell line RAW 264.7 was obtained from cryopreserved culture kindly provided by the Autonomous University of Queretaro, Mexico, which was originally from the ATCC (American Type Culture Collection). Macrophages were cultured in DMEM (Dulbecco's modified Eagle's medium) supplemented with 10% fetal bovine serum, 1% antibiotic-antimycotic, 1.5 g/L sodium bicarbonate, and 1 mL/L sodium pyruvate (1 mM) on a 60 mm plate and grown at 37°C and 5% CO_2_ in a humidified atmosphere [26].

#### 2.8.2. Effect of Sorghum Bran Extract on Cell Viability

RAW cells were cultured in 96-well plates at 1 × 10^4^ cells/well at 37°C and 5% CO_2_ for 24 h. Then, the culture medium was replaced by fresh medium in the absence (control viability 100%) and presence of different concentrations of extruded sorghum extract and nonextruded sorghum extract (extract equivalent to 4.3–10.1 mg sorghum/mL) previously lyophilized and dissolved in <1% DMSO. The culture was incubated for 24 h, and the medium was removed. The adhered cells were treated with 200 *μ*L of MTT dissolved in DMEM free of fetal bovine serum and incubated for 2 h. The transformation of MTT to formazan by the action of the enzyme succinate dehydrogenase was evaluated at 570 nm. Cell viability was expressed as a percentage, calculated by the following equation: % cell  viability = (absorbance of the sample/absorbance control cells) × 100 [26].

#### 2.8.3. Determination of Nitric Oxide Production

Nitric oxide production was measured according to the method previously reported by Nguyen et al. [26] with slight modifications. Briefly, RAW cells were cultured in 96-well plates at a density of 2.5 × 10^5^ cells/well at 37°C and 5% CO_2 _for 24 h. Subsequently, the production of nitric oxide (NO) in cells was induced with lipopolysaccharide (LPS) (1 *μ*g/mL) in the presence and absence of EB and NEB extracts for 24 h. NO production in the culture medium was assessed indirectly as nitrite by the Griess reaction. The supernatant medium (100 *μ*L) was mixed with 100 *μ*L of Griess reagent (1% sulfanilamide and 0.1% naphthylethylenediamine dihydrochloride) and incubated for 10 min. Absorbance was measured at 550 nm. The concentration of nitric oxide in the culture medium was determined based on a standard sodium nitrite curve.

### 2.9. Statistical Analysis

The effect of independent factors (moisture and temperature) and their interaction on the response variables was determined by ANOVA. Tukey's test was used for the comparison of the means. Statistical analyses were performed with the program JMP 5.0.1 (USA, SAS institute, Inc.). Values of *p* < 0.05 were accepted as statistically significant.

## 3. Results and Discussion

### 3.1. Total Phenolic and Phenolic Acid Content

Table 1 shows the total phenols and phenolic acid content of extruded and nonextruded sorghum bran. The extrusion process increased total phenol content in sorghum bran compared to nonextruded sorghum, particularly those extruded at 180°C and 20% moisture content (2.0222 ± 0.0157 versus 3.0729 ± 0.0187 mg GAE/g +52%).

The total hydroxycinnamic acids content increased in all extruded sorghum bran samples evaluated in this study compared to NEB. The EB samples treated at 180°C showed a higher total HCA content compared to the rest of the extruded samples. The amount of caffeic acid, *ρ*-coumaric acid, ferulic acid, and sinapic acid significantly increased after the extrusion process. Ferulic acid was the main phenolic acid found in the sorghum bran before and after the extrusion process. However, its concentration increased 2.7-fold with respect to NEB when heat treatment at 180°C was used.

The results obtained in this study, applying extrusion to sorghum bran, agree with those found by Zielinski et al. [19]. These authors observed an increase in the total phenolic and hydroxycinnamic acids, mainly ferulic and coumaric acids, in barley, rice, oats, and wheat as a result of extrusion at temperatures of 120°C, 160°C, and 200°C and 20% moisture. They suggested that heat treatment of cereals enhances the release of phenolic acids and their products from the cell walls. This last statement agrees with Ti et al. [27] who reported an increase of 12.6% in total phenolic content of rice bran as a consequence of the extrusion process. The release of phenol and other related compounds is a function of food matrix and extrusion conditions. Therefore, optimization of extrusion processes has to be established depending on the food matrix to have the highest release of bioactive compounds.

### 3.2. Antiradical Capacity

**Figure 1:**
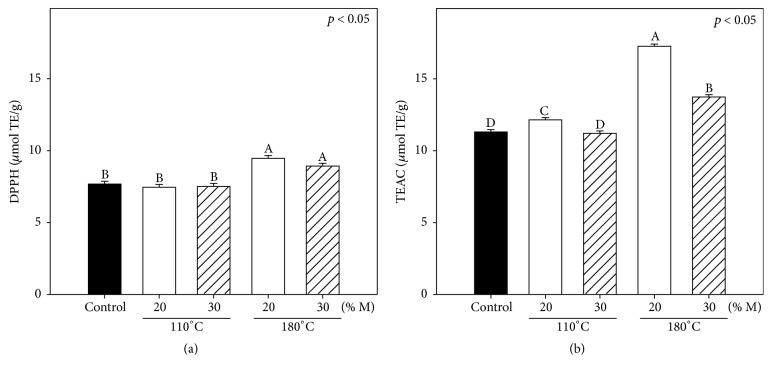
Antiradical capacity of sorghum bran before (control) and after extrusion processes: (a) DPPH and (b) TEAC. Each bar represents the mean of three replicates ± standard error. Different letters on bars represent significant differences (*p* < 0.05) between treatments including control.

The effects on the antiradical capacity of sorghum before and after the extrusion processes measured by the DPPH and TEAC assays are shown in Figure 1. We observed that the antiradical capacities in both assays (DPPH and TEAC) were higher (*p* < 0.05) for sorghum bran extruded at 180°C, with 9.5 ± 0.4 and 17.3 ± 0.4 *μ*g TE/g, than for nonextruded sorghum bran, with 7.7 ± 0.7 and 11.3 ± 0.4 *μ*g TE/g, respectively (Figures 1(a) and 1(b)). The antiradical capacity of extracts extruded at temperature of 110°C was lower (*p* < 0.05) than that of those extracts extruded at 180°C. This could be explained by the fact that temperatures above 170°C are sufficient to break down the chemical bond of lignin and ferulic acid [28] which could fragment the structure of arabinoxylans and consequently enhance the release of ferulic acid and increment the antiradical activity. Our results agreed with those reported by Ti et al. [27], where an increase of 19.7% in the antioxidant capacity of rice bran as a result of the extrusion process was observed.

**Figure 2:**
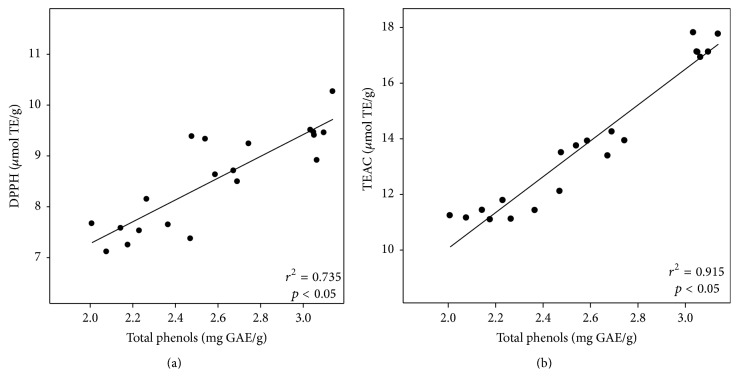
Correlations between the contents of total phenols in extruded sorghum bran and their antiradical capacity as determined by DPPH (a) and TEAC (b) assays.

To evaluate a possible association between changes in antioxidant capacity and total phenolic content (TPC), a correlation analysis was performed. A significant correlation was found between TPC and DPPH (*r* ^2^ = 0.735; *p* < 0.05) (Figure 2(a)) and between TPC and TEAC (*r* ^2^ = 0.915; *p* < 0.05) (Figure 2(b)). These findings suggest that total phenolic content is a good predictor of* in vitro* antiradical capacity. Shih et al. [29] reported a concomitant relationship between total phenolic content and antioxidant capacity (DPPH) in sweet potatoes after the extrusion process.

Additionally, a concomitant increase in antioxidant capacity and the content of phenolic acids, mainly ferulic acid, was previously reported in extruded rye [20]. The increase of phenolic compounds and antiradical capacity due to extrusion could be explained by the structural modification of the cell walls, where phenolic acids, such as ferulic and *ρ*-coumaric acids, are covalently linked to arabinoxylans favoring the release of these compounds. The increase in the efficacy of the extraction process can be accomplished by the modification of the bran matrix under conditions of high temperature, pressure, and shear [19, 27].

Extrusion processing of cereals provides advantages in terms of phenolic compound content and antioxidant capacity compared to the conventional wet cooking and soaking methods [11, 18].

In extruded corn flour, Mora-Rochin et al. [30] showed that the extrusion process has some advantages over the nixtamalization process. They evaluated the phenolic compound content and antioxidant capacity of corn tortillas and observed that tortillas made with extruded corn flour retain a higher content of total phenolic compounds, ferulic acid, and antioxidant capacity compared to tortillas prepared with nixtamalized corn flour in the traditional method. Nevertheless, Dlamini et al. [10] reported that porridge obtained from African sorghum by traditional methods had higher antioxidant capacity that that of products cooked by extrusion.

The extrusion temperature of 180°C was a determining factor in the achievement of a higher total phenol content. However, further studies focusing on combined processes may be necessary to increase the biological potential of sorghum bran. These differences in the content of phenolic compounds and antioxidant capacity of extruded cereals, and also their nutritional value, depend on the conditions used in the extrusion process and the chemical composition of the food matrix [21].

### 3.3. Nitric Oxide Production

**Figure 3:**
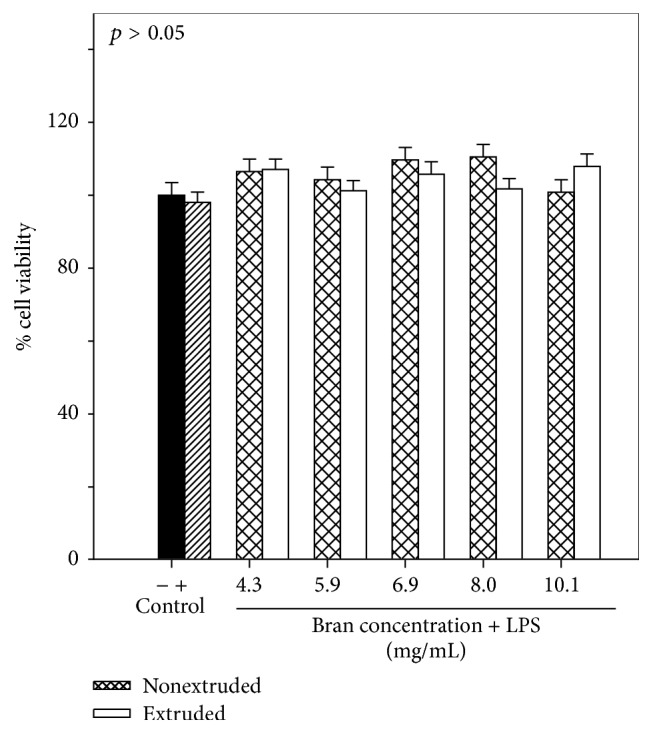
Cell viability (%) of RAW 264.7 cells treated with extruded sorghum bran (180°C/20% moisture) and nonextruded sorghum bran. Control (−) represents untreated cells and control (+) represents cells treated with LPS only. Each bar represents the mean of five replicates from three independent experiments ± standard error.

The inhibition of NO production by cell culture has been widely used as a biomarker to assess anti-inflammatory capacity because NO production is exacerbated by the action of the inducible nitric oxide synthase (iNOS), which is activated under conditions of oxidative stress, the presence of polysaccharides in Gram-negative bacteria, the tumor necrosis factor (TNF-*α*), and interleukin-1*β* which causes the activation of nuclear factor kappa B (NF-*κ*B) and the production of proinflammatory cytokines [31–34]. NO production by RAW 264.7 macrophages in the presence of extruded and nonextruded sorghum bran extracts was evaluated. For this assay, the extrusion treatment with the higher content of total phenols and antiradical capacity was selected (180°C/20% moisture). Prior to nitric oxide evaluation, the possible cytotoxic effects of sorghum bran phenolic extracts were measured using the MTT assay. When extruded or nonextruded sorghum bran extracts were added to LPS-activated RAW 264.7, no significant (*p* > 0.05) effects on the cell viability (%) of the RAW 264.7 cells at 4.3–10.1 mg sorghum bran/mL were observed (Figure 3).

**Figure 4:**
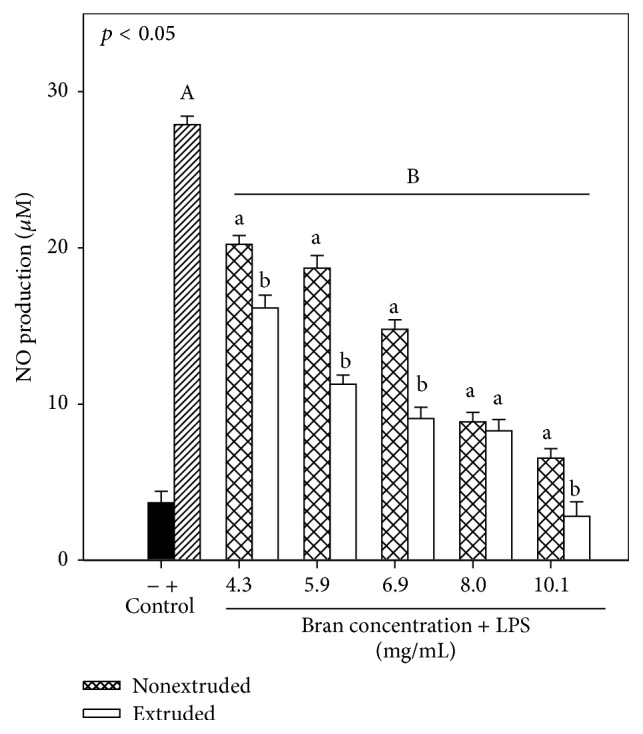
Nitric oxide production of RAW 264.7 cells treated with extruded sorghum bran at 180°C and 20% moisture and nonextruded sorghum bran. Control (−) represents untreated cell and control (+) represents cells treated with LPS only. Each bar represents the mean of five replicates from three independent experiments ± standard error. Bars with different letters in the same concentration are significantly different (*p* < 0.05). Capital letters represent significant differences (*p* < 0.05) between nonextruded and extruded treatments and positive control (LPS).

Figure 4 shows the effects of extruded or nonextruded sorghum bran on the production of nitric oxide by LPS-induced RAW 264.7 mouse macrophages. According to the concentrations of both extruded and nonextruded sorghum bran extracts selected for this study, it was observed that NO production was reduced significantly compared to the positive control (LPS-activated RAW 264.7). A dose-response effect on nitric oxide production in sorghum bran extracts evaluated was also observed. Using these results, the concentration of extract (extruded or nonextruded) was calculated, and the concentration at which there was 50% inhibition of nitric oxide production (EC_50_) was obtained, showing lower EC_50_ for extruded sorghum bran (5.23 mg/mL) than that of nonextruded sorghum bran (6.58 mg/mL).

With respect to nitric oxide production, it was found that bran sorghum subjected to the extrusion process showed less nitric oxide production (*p* < 0.05). Considering the maximum concentration of the sorghum extracts (10.1 mg/mL), the percentage of inhibition of NO production by RAW cells due to the presence of extruded sorghum bran extract was significantly higher (*p* < 0.05) than that of nonextruded sorghum bran extract (90.2 ± 1.9% versus 76.2 ± 1.3%). These results agree with those reported by Shim et al. [35] who evaluated the anti-inflammatory capacity of ethanol extracts of sorghum measured as inhibition of NO production in LPS-induced RAW 264.7 cells.

Hwang et al. [36] reported that chloroform extracts of sorghum showed a significantly higher inhibitory effect on the production of NO, iNOS, TNF*α*, and IL-6 in LPS-induced RAW cells compared to the inhibitory effects of corn and barley extracts. However, these studies only evaluated anti-inflammatory capacity in sorghum grain without thermal processes. As far as we know, this is the first time that the anti-inflammatory capacity of extruded sorghum bran has been evaluated* in vitro*.

Several studies of sorghum grain have reported an association between phenolic compounds and anti-inflammatory capacity. In this context, Burdette et al. [4] observed a correlation between the anti-inflammatory capacity of sorghum extracts and their phenolic compound content and antioxidant capacity. Hwang et al. [36] established a relationship between the anti-inflammatory capacity and the content of flavonoids. Previous studies have shown that white sorghum variety is poor in flavonoid content. Therefore, the content of phenolic compounds and the antioxidant capacity apparently is provided mainly by the phenolic acid derivatives of cinnamic acid [37, 38].

Previous reports have indicated that the antioxidant capacity of cereal extracts is due to the presence of cinnamic acids, which are able to inhibit the pathway of nuclear factor- (NF-) *κ*B. Kim et al. [39] evaluated the anti-inflammatory capacity of hydroxycinnamic acids isolated from corn bran in RAW 264.7 macrophages and observed inhibition of iNOS and NO production in connection to the NF-*κ*B pathway. Yun et al. [40] and Shin et al. [41] evaluated the effect of sinapic acid (40 to 160 *μ*M) and caffeic acid and its derivatives (25–100 *μ*M) on anti-inflammatory capacity and reported that the inhibitory effects were due to the suppression of iNOS, COX-2, TNF-*α*, and IL-1*β* expression through the effect of the NF-*κ*B pathway on RAW 264.7 macrophages. Other phenolic compounds such as quercetin and caffeic acid phenethyl ester also have been able to block the activation of NF-*κ*B and, as a consequence, inhibit the production of iNOS and NO [32].

## 4. Conclusions

Applying the extrusion process to sorghum bran increased total phenol and cinnamic acid contents, which positively affected the antioxidant capacity and the inhibition of LPS-induced nitric oxide production in RAW macrophages. The extrusion process could be a good alternative for processing sorghum bran to increase its functionality. This improvement of extruded sorghum bran can be beneficial for people with diseases related to oxidative stress and inflammation. Additional studies examining the increase in bioaccessibility of phenolic compounds of extruded sorghum bran are in progress.

## Figures and Tables

**Table 1:** Phenolic acid content and total phenols in sorghum bran extract before and after extrusion processes.

*T*°C	% M^*∗∗*^	Phenolic acid content (*μ*g/g)				Total HCAs^*∗∗∗*^	Total phenols (mg GAE/g)
		Caffeic	Coumaric	Ferulic	Sinapic		
Nonextruded		14.9 ± 0.3^c^^*∗*^	8.7 ± 0.2^e^	19.8 ± 0.2^c^	3.4 ± 0.1^d^	46.8 ± 0.5^d^	2.0222 ± 0.0157^d^
110	20	28.8 ± 0.2^a^	21.5 ± 0.3^a^	30.0 ± 0.9^b^	5.0 ± 0.1^c^	85.0 ± 0.7^b^	2.4068 ± 0.1079^c^
110	30	19.6 ± 0.2^b^	19.7 ± 0.6^b^	28.6 ± 0.3^b^	4.6 ± 0.0^c^	70.7 ± 2.3^c^	2.1336 ± 0.0516^d^
180	20	19.9 ± 0.8^b^	17.3 ± 0.2^c^	53.9 ± 1.6^a^	7.7 ± 0.2^a^	98.9 ± 1.3^a^	3.0729 ± 0.0187^a^
180	30	20.1 ± 0.9^b^	14.9 ± 0.3^d^	53.8 ± 1.1^a^	6.5 ± 0.2^b^	95.3 ± 2.4^a^	2.6192 ± 0.0101^b^

^*∗*^Each value represents the mean of three replicates ± standard error. Different letters within each column indicate significant differences (*p* < 0.05).

^*∗∗*^%  M: % moisture.

^*∗∗∗*^Total HCAs: total hydroxycinnamic acids.
